# Supplementary material for: Metabolomics for prediction of hypertension in pregnancy: a systematic review and meta-analysis protocol
Source: BMJ Open. 2020 Dec 29;10(12):e040652. doi: 10.1136/bmjopen-2020-040652 (PMC7778786; doi:10.1136/bmjopen-2020-040652)
Supplement: Author's manuscript [file bmjopen-2020-040652.draft_revisions.pdf]

BMJ Open is committed to open peer review. As part of this commitment we make the peer review history of every article we publish publicly available.

When an article is published we post the peer reviewers' comments and the authors' responses online. We also post the versions of the paper that were used during peer review. These are the versions that the peer review comments apply to.

The versions of the paper that follow are the versions that were submitted during the peer review process. They are not the versions of record or the final published versions. They should not be cited or distributed as the published version of this manuscript.

BMJ Open is an open access journal and the full, final, typeset and author-corrected version of record of the manuscript is available on our site with no access controls, subscription charges or pay-per-view fees (http://bmjopen.bmj.com).

If you have any questions on BMJ Open's open peer review process please email info.bmjopen@bmj.com

Metabolomics for prediction of hypertension in pregnancy: a systematic review and meta-analysis protocol

Journal:	*BMJ Open*
Manuscript ID	bmjopen-2020-040652
Article Type:	Protocol
Date Submitted by the Author:	19-May-2020

Complete List of Authors:	Mayrink, Jussara; Universidade Estadual de Campinas Faculdade de Ciencias Medicas<br>Leite, Debora; Campinas' State University, Department of Tocogynecology; Universidade Federal de Pernambuco,<br>Costa, Maria Laura<br>Cecatti, Jose; University of Campinas, Obstetrics and Gynecology

Keywords:	Hypertension < CARDIOLOGY, Prenatal diagnosis < OBSTETRICS, EPIDEMIOLOGY

I, the Submitting Author has the right to grant and does grant on behalf of all authors of the Work (as defined in the below author licence), an exclusive licence and/or a non-exclusive licence for contributions from authors who are: i) UK Crown employees; ii) where BMJ has agreed a CC-BY licence shall apply, and/or iii) in accordance with the terms applicable for US Federal Government officers or employees acting as part of their official duties; on a worldwide, perpetual, irrevocable, royalty-free basis to BMJ Publishing Group Ltd ("BMJ") its licensees and where the relevant Journal is co-owned by BMJ to the co-owners of the Journal, to publish the Work in this journal and any other BMJ products and to exploit all rights, as set out in our licence.

The Submitting Author accepts and understands that any supply made under these terms is made by BMJ to the Submitting Author unless you are acting as an employee on behalf of your employer or a postgraduate student of an affiliated institution which is paying any applicable article publishing charge ("APC") for Open Access articles. Where the Submitting Author wishes to make the Work available on an Open Access basis (and intends to pay the relevant APC), the terms of reuse of such Open Access shall be governed by a Creative Commons licence – details of these licences and which Creative Commons licence will apply to this Work are set out in our licence referred to above.

Other than as permitted in any relevant BMJ Author's Self Archiving Policies, I confirm this Work has not been accepted for publication elsewhere, is not being considered for publication elsewhere and does not duplicate material already published. I confirm all authors consent to publication of this Work and authorise the granting of this licence.

For peer review only

SYSTEMATIC REVIEW PROTOCOL

Metabolomics for prediction of hypertension in pregnancy: a systematic review and meta-analysis protocol

Jussara Mayrink[1,3] (jussaramayrink@gmail.com)

Debora F. B. Leite[2,3] (deborafariasleite@gmail.com)

Maria Laura Costa[3] (mlaura@unicamp.br)

José Guilherme Cecatti[3] (cecatti@unicamp.br)

1. Obstetric Unit; Department of Obstetrics and Gynecology, School of Medicine, Federal University of Minas Gerais, Belo Horizonte, Brazil.
2. Department of Obstetrics and Gynecology, Federal University of Pernambuco, Recife, Brazil.
3. Obstetric Unit; Department of Obstetrics and Gynecology, School of Medical Sciences, University of Campinas, Campinas, Brazil.

Correspondence:

Jose Guilherme Cecatti

Dept Obstet Gynecol

University of Campinas

Brazil

E-mail: cecatti@unicamp.br

For peer review only - http://bmjopen.bmj.com/site/about/guidelines.xhtml

Abstract

Introduction: hypertension is a very important cause of maternal morbidity and mortality worldwide, despite efforts on prevention. The lack of a tool to provide effective and early prediction of hypertension for a high-risk group may contribute to improve maternal and fetal outcomes. Metabolomics has figured out as a promised technology to contribute to the improvement on hypertension in pregnancy prediction. **Methods and analysis**: our primary outcome is hypertensive disorders of pregnancy. A detailed systematic literature search will be performed in electronic databases, using controlled terms 'preeclampsia', 'hypertensive disorders', 'metabolomics' and 'prediction' (and their variations). Studies from the latest twenty years will be included, except case reports, reviews, cross-sectional studies, letter to editors, expert opinions, commentaries papers or non-human research. If possible, we will perform a meta-analysis. Two peer reviewers will independently perform the search and in cases of discordance a third reviewer will be consulted. **Ethics and dissemination:** the results of this review will present the current use and performance of metabolomics for predicting gestational hypertension. Such data could potentially guide future studies and interventions to improve existing prediction models.

Strengths and limitations of this study

- Electronic search will cover the most important current available scientific databases for health research;
- There will not be a language restriction;
- Considering the complexity of Metabolomics technology and its methods, there would be a limitation to perform a quantitative synthesis.

Key words

Preeclampsia, pregnancy, hypertension, hypertensive disorders, hypertensive syndromes, metabolomics, metabolome

Prospero register number: CRD42018097409

For peer review only - http://bmjopen.bmj.com/site/about/guidelines.xhtml

Introduction

Hypertensive disorders in pregnancy consist of a group of conditions including preeclampsia, gestational hypertension, preeclampsia superimposed to chronic hypertension, white coat hypertension, masked hypertension and transient hypertension [1 2] and appear as the second cause of maternal death in the world according to a study performed by the World Health Organization between 2003 and 2009 [3]. Preeclampsia is the leading cause of maternal morbidity and mortality in Brazil and in several other low-and middle-income countries [4 5]. Its prevalence can vary according to the set of analyses, but the number ranges from 2 to 10% of all pregnancies [4]. Every year, around 80 thousand women die because of preeclampsia and its complications [6], despite potential prevention implemented by low-dose aspirin [7]. This intervention can represent a reduction rate of around 50% in the incidence of the early-onset preeclampsia cases, which developed preeclampsia before 34 weeks of gestation [7 8]. In this scenario, prediction of pregnant women under high-risk to develop preeclampsia is a key topic.

Some biomarkers have been proposed as earlier predictors (placental growth factor - PlGF, pregnancy-associated plasma protein A-PAPP-A) combined with clinical factors (pulsatility index of uterine arteries at Dopplervelocimetry exam, mean arterial blood pressure), showing different and sometimes conflicting detection rates [9-12]. These studies present limitations regarding the number of participants enrolled and heterogeneity to assess the prediction performance of those factors. Furthermore, the proposed prediction models from combining those factors outline better detection rates for early-onset preeclampsia cases compared to late-onset cases [13-15].

For peer review only

For peer review only - http://bmjopen.bmj.com/site/about/guidelines.xhtml

In the last decade, with the broad application of omics technologies, metabolomics has been pointed as a promising tool for the identification of early predictors for many health disturbances [16-18] and preeclampsia is one of them. Through metabolomics, it would be possible to identify metabolites involved in the final line of gene expression and a phenotypic signature in high resolution of the disease to be studied [19-21]. Studies have provided some insights about preeclampsia prediction through metabolites, belonging to different chemical classes and showing different performances [20-23]. In 2010, Kenny et al provided the initial knowledge on the topic, identifying 14 metabolites belonging to different chemical classes. When combined in an algorithm they showed a very good performance, with an Area under the Curve (AUC) of 0.94 in a discovery phase of the study and a detection rate of 77%, considering a false-positive rate of 10% [22]. It represents a very important tool option for prediction, especially with regard to cases of late-onset preeclampsia, which are the majority and the most difficult cases to predict [13-15]. Thus, in the sense of the inexistence of a systematic review protocol registered in this topic as well as a systematic review in progress or published, the main objective of this systematic review is to determine the accuracy of metabolomics for predicting hypertensive disorders of pregnancy.

Question formulation

In view of the social and economic implication of hypertensive disorders, their consequences to maternal and fetal lives worldwide and the lack of a useful screening test, in parallel to the increase of applicability of omics technologies, this systematic review will

For peer review only - http://bmjopen.bmj.com/site/about/guidelines.xhtml

be guided by this question: what is the performance of metabolomics for predicting gestational hypertensive disorders? It is in accordance with the PICO method [24] and associated with the search strategy provided a preliminary flow chart of studies as summarized in figure 1.

Methods and Analysis

Search Strategy

Electronic searches of literature will be carried out with these following databases: PubMed, EMBASE, Scopus, Web of Science, Latin America and Caribbean Health Sciences Literature (LILACS), Scientific Electronic Library Online (Scielo), Health Technology Assessment (HTA), Database of Abstracts of Reviews of Effects (DARE). We will include studies from the latest twenty years, considering that the vast majority of manuscripts on metabolomics are from this century. Our search strategy will combine terms with Boolean connectors related to the following categories: 1) hypertensive disorders, preeclampsia, pregnancy; 2) metabolomics, metabolome; and 3) screening, prediction. The Boolean connectors will be adapted according to the database used. We decided to use regular terms – not MESH or EMTREE terms – taking into account the number of databases consulted, in order to use always the same terms for all of them. In addition, we will search reference list of included articles, doing the backtracking of references. There will not be a language restriction. Before final publication, we will perform a new search in the databases in order to check if any study was published during the period of the systematic review elaboration. The databases exploration process and its results will follow the PRISMA Statement [25].

For peer review only - http://bmjopen.bmj.com/site/about/guidelines.xhtml

For peer review only

117 ***Study selection process***

118 After searching all sources of databases cited above, all the citations will be exported into EndNote® software. Firstly, two reviewers (JM and DFBL) will independently assess titles and abstracts. Only papers considered potentially relevant according to the inclusion criteria will be retrieved for further consideration. Cases of divergence will be analyzed by a third reviewer (MLC) who will do the final decision. A fourth reviewer (JGC) will check all procedures before approving the data extraction.

124 ***Study inclusion criteria***

125 Hypertensive disorders developed at any gestational age will be considered the domain studied. Previous other chronic conditions (diabetes, renal diseases, etc.) will be reported for stratification of analysis if the data allow for this. Original studies – including diagnostic studies - involving pregnant women are the inclusion criteria, and congenital malformation is the exclusion criteria.

130 ***Interventions/exposure***

131 Prediction of hypertensive disorders through metabolomics technologies is the intervention to be studied. The biomarker analysis should have been performed on samples taken before the hypertensive disorder diagnosis.

134 ***Design***

135 Our systematic review will include original studies (cohort or case control studies), including single or multiple pregnancies, as the studied population, and hypertensive disorders developed at any time of pregnancy, as the outcome of interest. We will exclude any studies that are: cross-sectional studies, case reports, editorials, letter to editors, commentaries,

For peer review only - http://bmjopen.bmj.com/site/about/guidelines.xhtml

expert opinions, any type of reviews, and experimental studies with animals, and when it is not possible to extract the data about the outcomes of interest.

Outcomes

We will include studies reporting outcomes of any hypertensive disorder developed during pregnancy. Our primary outcome is preeclampsia, defined as the onset of hypertension (a systolic blood pressure of 140 mmHg or more and/or a diastolic blood pressure of 90 mmHg or more) after 20 weeks of gestation, measured at least in two different occasions, combined with: (1) proteinuria (300 mg/day or at least 1g/L [1+] on dipstick testing or spot urine protein/creatinine>30mg/mmol [0.3mg/mg]) or (2) systemic complications or (3) uteroplacental dysfunction (fetal growth restriction) [1]. By systemic complications, we will consider:

- ✓ Hematological complications (thrombocytopenia- platelet count below 150,000/dL, disseminated intravascular coagulation, hemolysis);
- ✓ Hepatic dysfunction (elevated transaminases – at least twice upper limit of normal +- right upper quadrant or epigastric abdominal pain);
- ✓ Neurological dysfunction (examples include eclampsia, altered mental status, blindness, stroke or more commonly hyperreflexia when accompanied by clonus, severe headaches when accompanied by hyperreflexia, persistent visual scotomata;
- ✓ Renal dysfunction (creatinine > 1.2mg/dL);

Secondary outcomes include:

- ✓ Early-onset preeclampsia: when occurs before or at 33 weeks of gestation [26];
- ✓ Late-onset preeclampsia: when occurs at or after 34 weeks of gestation [26].

For peer review only - http://bmjopen.bmj.com/site/about/guidelines.xhtml

- ✓ Gestational hypertension: de novo development of high blood pressure after 20 weeks of gestation (a systolic blood pressure of 140 mmHg or more and/or a diastolic blood pressure of 90 mmHg or more), without any of the abnormalities that define preeclampsia as discussed above [1]
- ✓ White coat hypertension: it is demonstrated when a normal blood pressure is registered during 24 hours ambulatory monitoring in the first half of pregnancy [1]
- ✓ Preeclampsia superimposed on chronic hypertension: in a patient with high blood pressure predating the pregnancy, it is registered the occurrence of preeclampsia [1]
- ✓ Masked hypertension: is characterized by blood pressure that is normal at office or clinic but elevated at other times, most typically diagnosed by 24 hours ambulatory blood pressure monitoring [2]
- ✓ Transient gestational hypertension: is hypertension that arises in the second or third trimester. The hypertension is detected in the clinic but then settles with repeated blood pressure readings [2]

Data extraction

Data will be extracted through a standardized data compilation form in duplicate to avoid errors. The variables of interest from each included study are: authors, country, year of publication, study design, number of participants, preeclampsia prevalence, gestational age of recruitment, biological samples utilized, laboratory methods, metabolomics technology applied and metabolites. The metabolites will be matched with the Human Metabolome Database (HMDB) in order to check their biological function and chemical subclass. Missing

For peer review only

data will be requested from study authors. Pairs of data-extraction forms will be checked for discrepancies.

Quality Appraisal

The same two reviewers (JM and DFBL) who judged eligibility of papers will independently assess the risk of bias in included studies, but this time rating the methodological quality of the primary research. A third reviewer (MLC) will solve divergences when needed. Quality Assessment of Diagnostic Accuracy Studies (QUADAS-2) is the standard scale to be applied to access internal validity [27]. This tool is composed by four domains: patient selection, index test (metabolomics technique), reference standard (arterial blood pressure) and flow and timing of patient inclusion and follow up. Each domain is assessed in terms of risk of bias and the first three are assessed in terms of concerns regarding applicability. For each domain, every study will be labelled as "low", "high" or "unclear" risk of bias.

Funnel plots and sensitivity and cumulative analyses will be applied for detection of temporal trends and publication bias.

Strategy for data synthesis

In accordance with the Preferred Reporting Items for Systematic Reviews and Meta-Analysis (PRISMA), a flow diagram will be drawn [25]. Tables will show data regarding studies characteristics and risk of bias assessment for included and excluded studies. Narrative data will be analyzed and structured according to the outcomes: preeclampsia, gestational hypertension, transient gestational hypertension, white coat hypertension, masked hypertension. If possible, we are going to perform a subgroup analysis according to the metabolomics methods applied: gas or liquid chromatography, coupled with mass

For peer review only - http://bmjopen.bmj.com/site/about/guidelines.xhtml

spectrometry, or proton nuclear magnetic resonance. We also intend to perform a sensitivity analysis on the basis of early and late preeclampsia cases if sufficient studies will be found.

A meta-analysis will be performed (hierarchical summary receiver characteristic operating curve, HSROC) and accuracy measures will be calculated depending on data availability. Heterogeneity will also be assessed, through I-square test, Hotelling's T-squared test and Cochran's Q test.

Ethics and dissemination

Prediction of hypertensive disorders has been studied over the years with specific challenges. Among nulliparous for example, there is no history of previous events and a previous history of preeclampsia, is considered the most consistent predictive risk factor [28]. Another challenge to overcome is regarding to late-onset preeclampsia cases, which represent the majority of them. As cited above, the algorithms composed by biochemical and clinical factors showed better results with early onset cases of preeclampsia [13-14].

Metabolomics is a very complex technology and it has emerged as a possibility for prediction of adverse pregnancy outcomes [29-31]. The techniques employed are nuclear magnetic resonance spectroscopy, gas or liquid chromatography-mass spectrometry, Fourier transform infrared spectrometry and capillary electrophoresis [31]. Because of this complexity, results may be different concerning to the metabolites found. Consequently, generalizing results is also a challenge to overcome. This systematic review will contribute

For peer review only - http://bmjopen.bmj.com/site/about/guidelines.xhtml

to optimize the knowledge about the metabolites found in the studies and perhaps classify them according to HMDB, enabling quality translational research.

In addition, this systematic review will contribute to establish the current state of knowledge concerning the capacity of metabolomics to predict the occurrence of preeclampsia. Taking into account that this outcome involves relevant consequences for maternal and neonatal lives, the development of a tool that would predict preeclampsia is essential. Furthermore, the results of this systematic review could be used to guide future studies in this field. Once published, this systematic review will be free available in an open access scientific journal.

For peer review only

Patient and Public Involvement

Patients or the public were not involved in the design, or conduct, or reporting, or dissemination plans of our research proposal.

List of abbreviations

PlGF- placental growth factor

PAPP-A- pregnancy-associated plasma protein A

AUC- area under the curve

LILACS- Latin America and Caribbean Health Sciences Literature

Scielo- Scientific Electronic Library Online

HTA- Health Technology Assessment

DARE- Database of Abstracts of Reviews of Effects

HMDB- Human Metabolome Database

QUADAS-2- Quality Assessment of Diagnostic Accuracy Studies

PRISMA- Preferred Reporting Items for Systematic Reviews and Meta-Analysis

HSROC- Hierarchical summary receiver characteristic operating curve

For peer review only - http://bmjopen.bmj.com/site/about/guidelines.xhtml

References

1. Tranquilli AL, Dekker G, Magee L, Roberts J, Sibai BM, Steyn W, et al. The classification, diagnosis and management of the hypertensive disorders of pregnancy: A revised statement from the ISSHP. Pregnancy Hypertens. 2014;4(2):97-104.
2. Brown MA, Magee LA, Kenny LC, Karumanchi SA, McCarthy FP, Saito S, et al. The hypertensive disorders of pregnancy: ISSHP classification, diagnosis & management recommendations for international practice. Pregnancy Hypertens. 2018.
3. Abalos E, Cuesta C, Grosso AL, Chou D, Say L. Global and regional estimates of preeclampsia and eclampsia: a systematic review. Eur J Obstet Gynecol Reprod Biol. 2013;170(1):1-7.
4. Moussa HN, Arian SE, Sibai BM. Management of hypertensive disorders in pregnancy. Womens Health (Lond). 2014;10(4):385-404.
5. Lotufo FA, Parpinelli MA, Osis MJ, Surita FG, Costa ML, Cecatti JG. Situational analysis of facilitators and barriers to availability and utilization of magnesium sulfate for eclampsia and severe preeclampsia in the public health system in Brazil. BMC Pregnancy Childbirth. 2016;16:254.
6. Myers JE, Kenny LC, McCowan LM, Chan EH, Dekker GA, Poston L, et al. Angiogenic factors combined with clinical risk factors to predict preterm pre-eclampsia in nulliparous women: a predictive test accuracy study. BJOG. 2013;120(10):1215-23.
7. Rolnik DL, Wright D, Poon LC, O'Gorman N, Syngelaki A, de Paco Matallana C, et al. Aspirin versus Placebo in Pregnancies at High Risk for Preterm Preeclampsia. N Engl J Med. 2017;377(7):613-22.
8. Gasse C, Boutin A, Coté M, Chaillet N, Bujold E, Demers S. First-trimester mean arterial blood pressure and the risk of preeclampsia: The Great Obstetrical Syndromes (GOS) study. Pregnancy Hypertens. 2017.
9. Poon LC, Syngelaki A, Akolekar R, Lai J, Nicolaides KH. Combined screening for preeclampsia and small for gestational age at 11-13 weeks. Fetal Diagn Ther. 2013;33(1):16-27.
10. Poon LC, Nicolaides KH. First-trimester maternal factors and biomarker screening for preeclampsia. Prenat Diagn. 2014;34(7):618-27.
11. Cnossen JS, Vollebregt KC, de Vrieze N, ter Riet G, Mol BW, Franx A, et al. Accuracy of mean arterial pressure and blood pressure measurements in predicting pre-eclampsia: systematic review and meta-analysis. BMJ. 2008;336(7653):1117-20.
12. Kenny LC, Black MA, Poston L, Taylor R, Myers JE, Baker PN, et al. Early pregnancy prediction of preeclampsia in nulliparous women, combining clinical risk and biomarkers: the Screening for Pregnancy Endpoints (SCOPE) international cohort study. Hypertension. 2014;64(3):644-52.
13. Parra-Cordero M, Rodrigo R, Barja P, Bosco C, Rencoret G, Sepúlveda-Martinez A, et al. Prediction of early and late pre-eclampsia from maternal characteristics, uterine artery Doppler and markers of vasculogenesis during first trimester of pregnancy. Ultrasound Obstet Gynecol. 2013;41(5):538-44.
14. Kuc S, Koster MP, Franx A, Schielen PC, Visser GH. Maternal characteristics, mean arterial pressure and serum markers in early prediction of preeclampsia. PLoS One. 2013;8(5):e63546.
15. Rodriguez A, Tuuli MG, Odibo AO. First-, Second-, and Third-Trimester Screening for Preeclampsia and Intrauterine Growth Restriction. Clin Lab Med. 2016;36(2):331-51.
16. Ciborowski M, Teul J, Martin-Ventura JL, Egido J, Barbas C. Metabolomics with LC-QTOF-MS permits the prediction of disease stage in aortic abdominal aneurysm based on plasma metabolic fingerprint. PLoS One. 2012;7(2):e31982.

For peer review only - http://bmjopen.bmj.com/site/about/guidelines.xhtml

17. Boudonck KJ, Mitchell MW, Német L, Keresztes L, Nyska A, Shinar D, et al. Discovery of metabolomics biomarkers for early detection of nephrotoxicity. Toxicol Pathol. 2009;37(3):280-92.
18. Balls M. Future improvements: replacement in vitro methods. ILAR J. 2002;43 Suppl:S69-73.
19. Kenny LC. Metabolomics of preeclampsia. Pregnancy Hypertension. 2010;1:S2.
20. Odibo AO, Goetzinger KR, Odibo L, Cahill AG, Macones GA, Nelson DM, et al. First-trimester prediction of preeclampsia using metabolomic biomarkers: A discovery phase study. Prenatal Diagnosis. 2011;31(10):990-4.
21. Bahado-Singh RO, Akolekar R, Mandal R, Dong E, Xia J, Kruger M, et al. Metabolomics and first-trimester prediction of early-onset preeclampsia. Journal of Maternal-Fetal and Neonatal Medicine. 2012;25(10):1840-7.
22. Kenny LC, Broadhurst DI, Dunn W, Brown M, North RA, McCowan L, et al. Robust early pregnancy prediction of later preeclampsia using metabolomic biomarkers. Hypertension. 2010;56(4):741-9.
23. Kuc S, Koster MP, Pennings JL, Hankemeier T, Berger R, Harms AC, et al. Metabolomics profiling for identification of novel potential markers in early prediction of preeclampsia. PLoS One. 2014;9(5):e98540.
24. Wright RW, Brand RA, Dunn W, Spindler KP. How to write a systematic review. Clin Orthop Relat Res. 2007;455:23-9.
25. Liberati A, Altman DG, Tetzlaff J, Mulrow C, Gøtzsche PC, Ioannidis JP, et al. The PRISMA statement for reporting systematic reviews and meta-analyses of studies that evaluate healthcare interventions: explanation and elaboration. BMJ. 2009;339:b2700.
26. Lisonkova S, Joseph KS. Incidence of preeclampsia: risk factors and outcomes associated with early- versus late-onset disease. Am J Obstet Gynecol. 2013;209(6):544.e1-.e12.
27. Whiting PF, Rutjes AW, Westwood ME, Mallett S, Deeks JJ, Reitsma JB, et al. QUADAS-2: a revised tool for the quality assessment of diagnostic accuracy studies. Ann Intern Med. 2011;155(8):529-36.
28. Poon LC, Kametas NA, Chelemen T, Leal A, Nicolaides KH. Maternal risk factors for hypertensive disorders in pregnancy: a multivariate approach. J Hum Hypertens. 2010;24(2):104-10.
29. Austdal M, Skråstad RB, Gundersen AS, Austgulen R, Iversen AC, Bathen TF. Metabolomic biomarkers in serum and urine in women with preeclampsia. PLoS One. 2014;9(3):e91923.
30. Austdal M, Tangerås LH, Skråstad RB, Salvesen KÅ, Austgulen R, Iversen AC, et al. First trimester urine and serum metabolomics for prediction of preeclampsia and gestational hypertension: A prospective screening study. International Journal of Molecular Sciences. 2015;16(9):21520-38.
31. Dessì A, Marincola FC, Fanos V. Metabolomics and the great obstetrical syndromes - GDM, PET, and IUGR. Best Practice and Research: Clinical Obstetrics and Gynaecology. 2015;29(2):156-64.

For peer review only - http://bmjopen.bmj.com/site/about/guidelines.xhtml

Acknowledgements

This is a modified version of the article that was part of the PhD thesis of Jussara de Souza Mayrink Novais presented to the Postgraduate Program on Obstetrics and Gynecology from the School of Medical Sciences of the University of Campinas, Brazil, under the tutorial of Jose Guilherme Cecatti and Maria Laura Costa on December 13th 2018.

Authors' contributions

JM worked out the protocol, developed searches and data management, will participate in selection, inclusion, quality assessment and data extraction of papers. DFBL helped working out the protocol and will participate in selection, inclusion, quality assessment and data extraction as well. MLC and JGC helped working out the protocol, and MLC will solve any disagreement concerning to the selected papers. JGC will supervise all the development of the systematic review. All authors read and approved this final manuscript.

Funding

This study is a sub product of the study "Preterm SAMBA" which was jointly financed by the Brazilian CNPq (CNPq, Grant 401636/2013-5) and the Bill and Melinda Gates Foundation (Grant OPP1107597).

Competing interests

The author(s) declare that they have not competing interests.

Word count: 1973 words

Figure Legend

Figure 1. Flow chart of studies identified to be included in the systematic review.

For peer review only

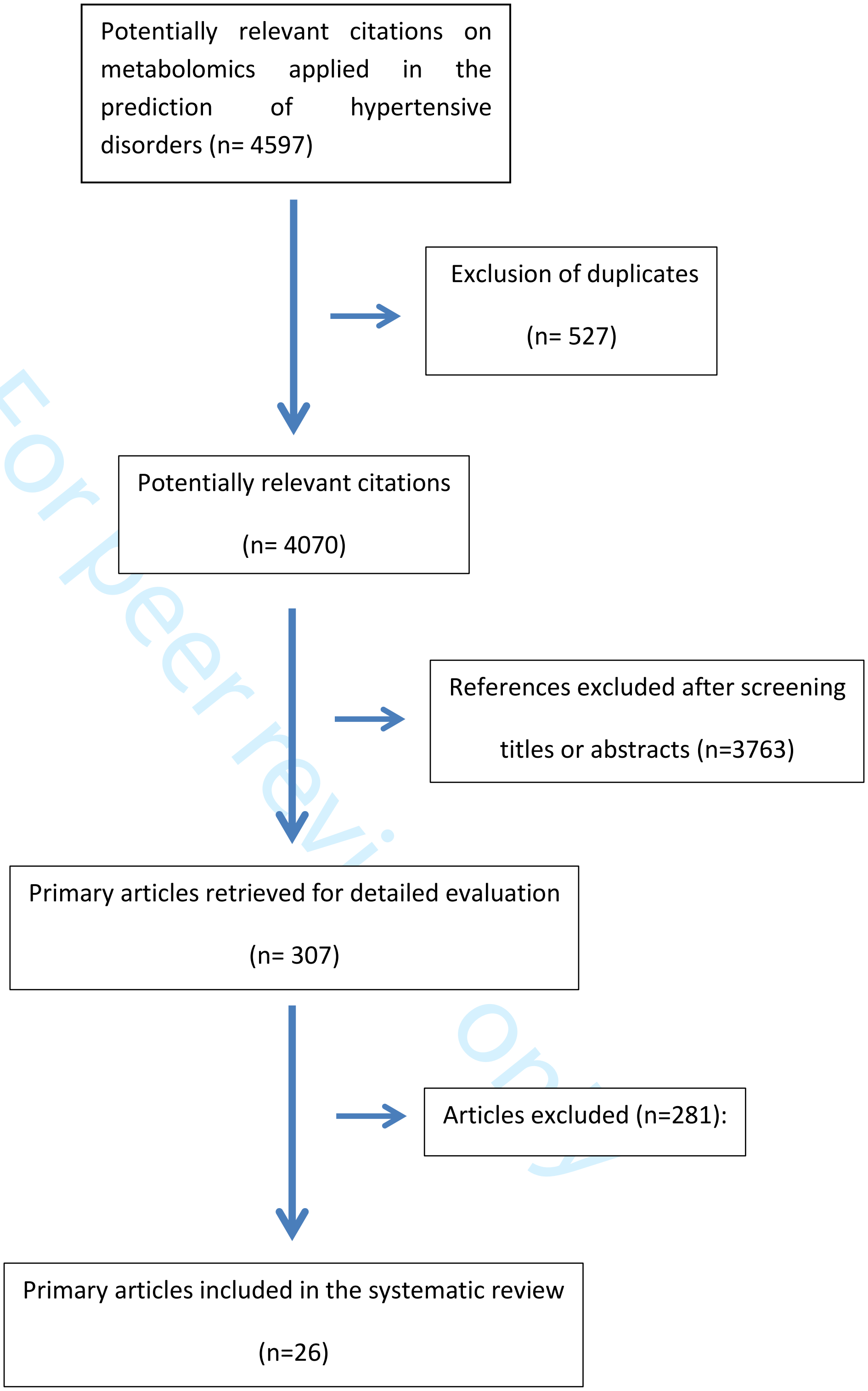

For peer review only

PRISMA-P 2015 Checklist

This checklist has been adapted for use with systematic review protocol submissions to BioMed Central journals from Table 3 in Moher D et al: Preferred reporting items for systematic review and meta-analysis protocols (PRISMA-P) 2015 statement. *Systematic Reviews* 2015 **4**:1

An Editorial from the Editors-in-Chief of *Systematic Reviews* details why this checklist was adapted - **Moher D, Stewart L & Shekelle P**: Implementing PRISMA-P: recommendations for prospective authors. *Systematic Reviews* 2016 **5**:15

Section/topic	#	Checklist item	Information reported Yes	No	Line number(s)
**ADMINISTRATIVE INFORMATION**					
**Title**					
Identification	1a	Identify the report as a protocol of a systematic review	x		3
Update	1b	If the protocol is for an update of a previous systematic review, identify as such		x	
**Registration**	2	If registered, provide the name of the registry (e.g., PROSPERO) and registration number in the Abstract	x		52
**Authors**					
Contact	3a	Provide name, institutional affiliation, and e-mail address of all protocol authors; provide physical mailing address of corresponding author	x		5,6,7,8
Contributions	3b	Describe contributions of protocol authors and identify the guarantor of the review	x		347-353
**Amendments**	4	If the protocol represents an amendment of a previously completed or published protocol, identify as such and list changes; otherwise, state plan for documenting important protocol amendments		x	
**Support**					
Sources	5a	Indicate sources of financial or other support for the review	x		344-345
Sponsor	5b	Provide name for the review funder and/or sponsor	x		344-345
Role of sponsor/funder	5c	Describe roles of funder(s), sponsor(s), and/or institution(s), if any, in developing the protocol		x	
**INTRODUCTION**					
**Rationale**	6	Describe the rationale for the review in the context of what is already known	x		54-91

For peer review only - http://bmjopen.bmj.com/site/about/guidelines.xhtml

BioMed Central The Open Access Publisher

Section/topic	#	Checklist item	Information reported: Yes	Information reported: No	Line number(s)
**Objectives**	7	Provide an explicit statement of the question(s) the review will address with reference to participants, interventions, comparators, and outcomes (PICO)	x		94-99
**METHODS**					
**Eligibility criteria**	8	Specify the study characteristics (e.g., PICO, study design, setting, time frame) and report characteristics (e.g., years considered, language, publication status) to be used as criteria for eligibility for the review	x		107-109; 128-132; 140-143
**Information sources**	9	Describe all intended information sources (e.g., electronic databases, contact with study authors, trial registers, or other grey literature sources) with planned dates of coverage	x		104-107
**Search strategy**	10	Present draft of search strategy to be used for at least one electronic database, including planned limits, such that it could be repeated	x		109-114
STUDY RECORDS					
Data management	11a	Describe the mechanism(s) that will be used to manage records and data throughout the review	x		121-122
Selection process	11b	State the process that will be used for selecting studies (e.g., two independent reviewers) through each phase of the review (i.e., screening, eligibility, and inclusion in meta-analysis)	x		122-126
Data collection process	11c	Describe planned method of extracting data from reports (e.g., piloting forms, done independently, in duplicate), any processes for obtaining and confirming data from investigators	x		184-191
**Data items**	12	List and define all variables for which data will be sought (e.g., PICO items, funding sources), any pre-planned data assumptions and simplifications		x	
**Outcomes and prioritization**	13	List and define all outcomes for which data will be sought, including prioritization of main and additional outcomes, with rationale	x		145-180
**Risk of bias in individual studies**	14	Describe anticipated methods for assessing risk of bias of individual studies, including whether this will be done at the outcome or study level, or both; state how this information will be used in data synthesis	x		193-204
DATA					
**Synthesis**	15a	Describe criteria under which study data will be quantitatively synthesized		x	
	15b	If data are appropriate for quantitative synthesis, describe planned summary measures, methods of handling data, and methods of combining data from studies, including any planned exploration of consistency (e.g., I^2, Kendall's tau)		x	
	15c	Describe any proposed additional analyses (e.g., sensitivity or subgroup analyses, meta-regression)		x	
	15d	If quantitative synthesis is not appropriate, describe the type of summary planned	x		206-219

For peer review only

BioMed Central The Open Access Publisher

For peer review only - http://bmjopen.bmj.com/site/about/guidelines.xhtml

Section/topic	#	Checklist item	Information reported		Line number(s)
			Yes	No	
**Meta-bias(es)**	16	Specify any planned assessment of meta-bias(es) (e.g., publication bias across studies, selective reporting within studies)	☐	x☐	
**Confidence in cumulative evidence**	17	Describe how the strength of the body of evidence will be assessed (e.g., GRADE)	☐	x☐	

For peer review only

BioMed Central The Open Access Publisher

BMJ Open

Metabolomics for prediction of hypertension in pregnancy: a systematic review and meta-analysis protocol

Journal:	*BMJ Open*
Manuscript ID	bmjopen-2020-040652.R1
Article Type:	Protocol
Date Submitted by the Author:	23-Nov-2020
Complete List of Authors:	Mayrink, Jussara; Universidade Estadual de Campinas Faculdade de Ciencias Medicas<br>Leite, Debora; State University of Campinas, Department of Tocogynecology; Universidade Federal de Pernambuco,<br>Costa, Maria Laura<br>Cecatti, Jose; State University of Campinas, Obstetrics and Gynecology
**Primary Subject Heading**:	Obstetrics and gynaecology
Secondary Subject Heading:	Epidemiology
Keywords:	Hypertension < CARDIOLOGY, Prenatal diagnosis < OBSTETRICS, EPIDEMIOLOGY

SCHOLARONE™
Manuscripts

I, the Submitting Author has the right to grant and does grant on behalf of all authors of the Work (as defined in the below author licence), an exclusive licence and/or a non-exclusive licence for contributions from authors who are: i) UK Crown employees; ii) where BMJ has agreed a CC-BY licence shall apply, and/or iii) in accordance with the terms applicable for US Federal Government officers or employees acting as part of their official duties; on a worldwide, perpetual, irrevocable, royalty-free basis to BMJ Publishing Group Ltd ("BMJ") its licensees and where the relevant Journal is co-owned by BMJ to the co-owners of the Journal, to publish the Work in this journal and any other BMJ products and to exploit all rights, as set out in our licence.

The Submitting Author accepts and understands that any supply made under these terms is made by BMJ to the Submitting Author unless you are acting as an employee on behalf of your employer or a postgraduate student of an affiliated institution which is paying any applicable article publishing charge ("APC") for Open Access articles. Where the Submitting Author wishes to make the Work available on an Open Access basis (and intends to pay the relevant APC), the terms of reuse of such Open Access shall be governed by a Creative Commons licence – details of these licences and which Creative Commons licence will apply to this Work are set out in our licence referred to above.

Other than as permitted in any relevant BMJ Author's Self Archiving Policies, I confirm this Work has not been accepted for publication elsewhere, is not being considered for publication elsewhere and does not duplicate material already published. I confirm all authors consent to publication of this Work and authorise the granting of this licence.

For peer review only

SYSTEMATIC REVIEW PROTOCOL

Metabolomics for prediction of hypertension in pregnancy: a systematic review and meta-analysis protocol

Jussara Mayrink[1,3] (jussaramayrink@gmail.com)

Debora F. B. Leite[2,3] (deborafariasleite@gmail.com)

Maria Laura Costa[3] (mlaura@unicamp.br)

José Guilherme Cecatti[3] (cecatti@unicamp.br)

1. Obstetric Unit; Department of Obstetrics and Gynecology, School of Medicine, Federal University of Minas Gerais, Belo Horizonte, Brazil.
2. Department of Obstetrics and Gynecology, Federal University of Pernambuco, Recife, Brazil.
3. Obstetric Unit; Department of Obstetrics and Gynecology, School of Medical Sciences, University of Campinas, Campinas, Brazil.

Correspondence:

Jose Guilherme Cecatti

Dept Obstet Gynecol

University of Campinas

Brazil

E-mail: cecatti@unicamp.br

For peer review only

For peer review only - http://bmjopen.bmj.com/site/about/guidelines.xhtml

Abstract

Introduction: hypertension is a very important cause of maternal morbidity and mortality worldwide, despite efforts on prevention. The lack of a tool to provide effective and early prediction of hypertension for a high-risk group may contribute to improving maternal and fetal outcomes. Metabolomics has figured out as a promised technology to contribute to the improvement of hypertension in pregnancy prediction. **Methods and analysis**: our primary outcome is hypertensive disorders of pregnancy. A detailed systematic literature search will be performed in electronic databases PubMed, EMBASE, Scopus, Web of Science, Latin America and Caribbean Health Sciences Literature, Scientific Electronic Library Online, Health Technology Assessment, and Database of Abstracts of Reviews of Effects using controlled terms 'preeclampsia', 'hypertensive disorders', 'metabolomics' and 'prediction' (and their variations). Studies from the latest twenty years will be included, except case reports, reviews, cross-sectional studies, letter to editors, expert opinions, commentaries papers or non-human research. If possible, we will perform a meta-analysis. Two peer reviewers will independently perform the search and in cases of discordance, a third reviewer will be consulted. **Ethics and dissemination:** as a systematic review, ethics approval is not required. The results of this review will present the current use and performance of metabolomics for predicting gestational hypertension. Such data could potentially guide future studies and interventions to improve existing prediction models.

Strengths and limitations of this study

- Electronic search will cover the most important current available scientific databases for health research;
- There will not be a language restriction;
- Considering the complexity of Metabolomics technology and its methods, there would be a limitation to perform a quantitative synthesis.

Keywords

For peer review only - http://bmjopen.bmj.com/site/about/guidelines.xhtml

Preeclampsia, pregnancy, hypertension, hypertensive disorders, hypertensive syndromes, metabolomics, metabolome

Prospero register number: CRD42018097409

Introduction

Hypertensive disorders in pregnancy consist of a group of conditions including preeclampsia, gestational hypertension, preeclampsia superimposed to chronic hypertension, white coat hypertension, masked hypertension and transient hypertension [1 2] and appear as the second cause of maternal death in the world according to a study performed by the World Health Organization between 2003 and 2009 [3]. Preeclampsia is the leading cause of maternal morbidity and mortality in Brazil and several other low-and middle-income countries [4 5]. Its prevalence can vary according to the set of analyses, but the number ranges from 2 to 10% of all pregnancies [4]. Every year, around 70 thousand women die because of preeclampsia and its complications [3], despite potential prevention implemented by low-dose aspirin [6,7]. This intervention can represent a reduction rate of around 50% in the incidence of the early-onset preeclampsia cases, which developed preeclampsia before 34 weeks of gestation [7 8]. In this scenario, the prediction of pregnant women under high-risk to develop preeclampsia is a key topic.

Some biomarkers have been proposed as earlier predictors (placental growth factor - PIGF, pregnancy-associated plasma protein A-PAPP-A) combined with clinical factors (pulsatility index of uterine arteries at Dopplervelocimetry exam, mean arterial blood pressure) in models with different detection and false-positive rates [9-12]. These studies present limitations regarding the number of participants enrolled and heterogeneity to

For peer review only - http://bmjopen.bmj.com/site/about/guidelines.xhtml

assess the prediction performance of those factors. Furthermore, the proposed prediction models from combining those factors outline better detection rates for early-onset preeclampsia cases compared to late-onset cases [13-15].

In the last decade, with the broad application of omics technologies, metabolomics has been pointed as a promising tool for the identification of early predictors for many health disturbances [16-18] and preeclampsia is one of them. Through metabolomics, it would be possible to identify metabolites involved in the final line of gene expression and a phenotypic signature in high resolution of the disease to be studied [19-21]. Studies have provided some insights about preeclampsia prediction through metabolites, belonging to different chemical classes and showing different performances [20-23]. In 2010, Kenny et al provided the initial knowledge on the topic, identifying 14 metabolites belonging to different chemical classes. When combined in an algorithm they showed a very good performance, with an Area under the Curve (AUC) of 0.94 in a discovery phase of the study and a detection rate of 77%, considering a false-positive rate of 10% [22]. It represents a very important tool option for prediction, especially concerning cases of late-onset preeclampsia, which are the majority and the most difficult cases to predict [13-15]. Thus, in the sense of the inexistence of a systematic review protocol registered in this topic as well as a systematic review in progress or published, the main objective of this systematic review is to determine the accuracy of metabolomics for predicting hypertensive disorders of pregnancy.

Question formulation

For peer review only - http://bmjopen.bmj.com/site/about/guidelines.xhtml

Because of the social and economic implication of hypertensive disorders, their consequences to maternal and fetal lives worldwide and the lack of a useful screening test, in parallel to the increase of applicability of omics technologies, this systematic review will be guided by this question: what is the performance of metabolomics for predicting gestational hypertensive disorders? It is following the PICO method [24] and associated with the search strategy provided a preliminary flow chart of studies as summarized in figure 1.

Methods and Analysis

Search Strategy

Electronic searches of literature will be carried out with these following databases: PubMed, EMBASE, Scopus, Web of Science, Latin America and Caribbean Health Sciences Literature (LILACS), Scientific Electronic Library Online (Scielo), Health Technology Assessment (HTA), Database of Abstracts of Reviews of Effects (DARE). We will include studies from the latest twenty years, considering that the vast majority of manuscripts on metabolomics are from this century. Our search strategy will combine terms with Boolean connectors related to the following categories: 1) hypertensive disorders, preeclampsia, pregnancy; 2) metabolomics, metabolome; and 3) screening, prediction. The Boolean connectors will be adapted according to the database used. We decided to use regular terms – not MESH or EMTREE terms – taking into account the number of databases consulted, to use always the same terms for all of them. Also, we will search reference list of included articles, doing the backtracking of references. There will not be a language restriction. Before final publication, we will perform a new search in the databases to check if any study was published during

For peer review only

For peer review only - http://bmjopen.bmj.com/site/about/guidelines.xhtml

the period of the systematic review elaboration. The databases exploration process and its results will follow the PRISMA Statement [25].

Study selection process

After searching all sources of databases cited above, all the citations will be exported into EndNote® software. Firstly, two reviewers (JM and DFBL) will independently assess titles and abstracts. Only papers considered potentially relevant according to the inclusion criteria will be retrieved for further consideration. Cases of divergence will be analyzed by a third reviewer (MLC) who will do the final decision. A fourth reviewer (JGC) will check all procedures before approving the data extraction.

Study inclusion criteria

Hypertensive disorders developed at any gestational age will be considered the domain studied. Previous other chronic conditions (diabetes, renal diseases, etc.) will be reported for stratification of analysis if the data allow for this. Original studies – including diagnostic studies - involving pregnant women are the inclusion criteria, and congenital malformation is the exclusion criteria.

Interventions/exposure

Prediction of hypertensive disorders through metabolomics technologies is the intervention to be studied. The biomarker analysis should have been performed on samples taken before the hypertensive disorder diagnosis.

Design

Our systematic review will include original studies (cohort or case-control studies), including single or multiple pregnancies, as the studied population, and hypertensive disorders

For peer review only

developed at any time of pregnancy, as the outcome of interest. We will exclude any studies that are: cross-sectional studies, case reports, editorials, letter to editors, commentaries, expert opinions, any type of reviews, and experimental studies with animals, and when it is not possible to extract the data about the outcomes of interest.

Outcomes

We will include studies reporting outcomes of any hypertensive disorder developed during pregnancy. Our primary outcome is preeclampsia, defined as the onset of hypertension (systolic blood pressure of 140 mmHg or more and/or diastolic blood pressure of 90 mmHg or more) after 20 weeks of gestation, measured at least in two different occasions, combined with (1) proteinuria (300 mg/day or at least 1g/L [1+] on dipstick testing or spot urine protein/creatinine>30mg/mmol [0.3mg/mg]) or (2) systemic complications or (3) uteroplacental dysfunction (fetal growth restriction) [1]. By systemic complications, we will consider:

- ✓ Hematological complications (thrombocytopenia- platelet count below 150,000/dL, disseminated intravascular coagulation, hemolysis);
- ✓ Hepatic dysfunction (elevated transaminases – at least twice upper limit of normal +- right upper quadrant or epigastric abdominal pain);
- ✓ Neurological dysfunction (examples include eclampsia, altered mental status, blindness, stroke or more commonly hyperreflexia when accompanied by clonus, severe headaches when accompanied by hyperreflexia, persistent visual scotomata;
- ✓ Renal dysfunction (creatinine > 1.2mg/dL);

Secondary outcomes include:

For peer review only - http://bmjopen.bmj.com/site/about/guidelines.xhtml

- ✓ Early-onset preeclampsia: when occurs before or at 33 weeks of gestation [26];
- ✓ Late-onset preeclampsia: when occurs at or after 34 weeks of gestation [26].
- ✓ Gestational hypertension: de novo development of high blood pressure after 20 weeks of gestation (systolic blood pressure of 140 mmHg or more and/or diastolic blood pressure of 90 mmHg or more), without any of the abnormalities that define preeclampsia as discussed above [1]
- ✓ Whitecoat hypertension: it is demonstrated when normal blood pressure is registered during 24 hours ambulatory monitoring in the first half of pregnancy [1]
- ✓ Preeclampsia superimposed on chronic hypertension: in a patient with high blood pressure predating the pregnancy, it is registered the occurrence of preeclampsia [1]
- ✓ Masked hypertension: is characterized by blood pressure that is normal at office or clinic but elevated at other times, most typically diagnosed by 24 hours ambulatory blood pressure monitoring [2]
- ✓ Transient gestational hypertension is hypertension that arises in the second or third trimester. The hypertension is detected in the clinic but then settles with repeated blood pressure readings [2]

Data extraction

Data will be extracted through a standardized data compilation form in duplicate to avoid errors. The variables of interest from each included study are: authors, country, year of publication, study design, number of participants, preeclampsia prevalence, gestational age of recruitment, biological samples utilized, laboratory methods, metabolomics technology

For peer review only - http://bmjopen.bmj.com/site/about/guidelines.xhtml

applied and metabolites. The metabolites will be matched with the Human Metabolome Database (HMDB) to check their biological function and chemical subclass. Missing data will be requested from study authors. Pairs of data-extraction forms will be checked for discrepancies.

Quality Appraisal

The same two reviewers (JM and DFBL) who judged eligibility of papers will independently assess the risk of bias in included studies, but this time rating the methodological quality of the primary research. A third reviewer (MLC) will solve divergences when needed. Quality Assessment of Diagnostic Accuracy Studies (QUADAS-2) is the standard scale to be applied to access internal validity [27]. This tool is composed of four domains: patient selection, index test (metabolomics technique), reference standard (arterial blood pressure) and flow and timing of patient inclusion and follow up. Each domain is assessed in terms of risk of bias and the first three are assessed in terms of concerns regarding applicability. For each domain, every study will be labelled as “low”, “high” or “unclear” risk of bias.

Funnel plots and sensitivity and cumulative analyses will be applied for the detection of temporal trends and publication bias.

Strategy for data synthesis

Following the Preferred Reporting Items for Systematic Reviews and Meta-Analysis (PRISMA), a flow diagram will be drawn [25]. Tables will show data regarding studies characteristics and risk of bias assessment for included and excluded studies. Narrative data will be analyzed and structured according to the outcomes: preeclampsia, gestational hypertension, transient gestational hypertension, white coat hypertension, masked

For peer review only - http://bmjopen.bmj.com/site/about/guidelines.xhtml

hypertension. If possible, we are going to perform subgroup analysis according to the metabolomics methods applied: gas or liquid chromatography, coupled with mass spectrometry, or proton nuclear magnetic resonance, and based on ethnic group and the severity of the hypertensive disease. We also intend to perform a sensitivity analysis based on early and late preeclampsia cases if sufficient studies will be found.

A meta-analysis will be performed (hierarchical summary receiver characteristic operating curve, HSROC) and accuracy measures will be calculated depending on data availability. If a meta-analysis will be possible, considering the limitations imposed by data heterogeneity and drawings of the vast majority of studies, we intend to use RevMan software. Taking into account that the studies involve the frequency of metabolites and occurrence of preeclampsia, we are going to use a fixed-effect model or random- effect model, depending on the heterogeneity found. Heterogeneity will also be assessed, through the l-square test, Hotelling's T-squared test and Cochran's Q test.

Ethics and dissemination

Prediction of hypertensive disorders has been studied over the years with specific challenges. Among nulliparous for example, there is no history of previous events and a previous history of preeclampsia, is considered the most consistent predictive risk factor [28]. Another challenge to overcome is regarding late-onset preeclampsia cases, which represent the majority of them. As cited above, the algorithms composed by biochemical and clinical factors showed better results with early-onset cases of preeclampsia [13-14].

For peer review only - http://bmjopen.bmj.com/site/about/guidelines.xhtml

Metabolomics is a very complex technology and it has emerged as a possibility for prediction of adverse pregnancy outcomes [29-31]. The techniques employed are nuclear magnetic resonance spectroscopy, gas or liquid chromatography-mass spectrometry, Fourier transforms infrared spectrometry and capillary electrophoresis [31]. Because of this complexity, results may be different concerning the metabolites found. Consequently, generalizing results is also a challenge to overcome. This systematic review will contribute to optimize the knowledge about the metabolites found in the studies and perhaps classify them according to HMDB, enabling quality translational research.

Besides, this systematic review will contribute to establishing the current state of knowledge concerning the capacity of metabolomics to predict the occurrence of preeclampsia. Taking into account that this outcome involves relevant consequences for maternal and neonatal lives, the development of a tool that would predict preeclampsia is essential. Furthermore, the results of this systematic review could be used to guide future studies in this field. Once published, this systematic review will be freely available in an open-access scientific journal.

Patient and Public Involvement

Patients or the public were not involved in the design, or conduct, or reporting, or dissemination plans of our research proposal.

List of abbreviations

PlGF- placental growth factor

PAPP-A- pregnancy-associated plasma protein A

AUC- area under the curve

LILACS- Latin America and Caribbean Health Sciences Literature

For peer review only - http://bmjopen.bmj.com/site/about/guidelines.xhtml

Scielo- Scientific Electronic Library Online

HTA- Health Technology Assessment

DARE- Database of Abstracts of Reviews of Effects

HMDB- Human Metabolome Database

QUADAS-2- Quality Assessment of Diagnostic Accuracy Studies

PRISMA- Preferred Reporting Items for Systematic Reviews and Meta-Analysis

HSROC- Hierarchical summary receiver characteristic operating curve

Acknowledgements

This is a modified version of the article that was part of the PhD thesis of Jussara de Souza Mayrink Novais presented to the Postgraduate Program on Obstetrics and Gynecology from the School of Medical Sciences of the University of Campinas, Brazil, under the tutorial of Jose Guilherme Cecatti and Maria Laura Costa on December 13th 2018.

Authors' contributions

JM worked out the protocol, developed searches and data management, will participate in the selection, inclusion, quality assessment and data extraction of papers. DFBL helped working out the protocol and will participate in selection, inclusion, quality assessment and data extraction as well. MLC and JGC helped working out the protocol, and MLC will solve any disagreement concerning the selected papers. JGC will supervise all the development of the systematic review. All authors read and approved this final manuscript.

Funding

This study is a sub-product of the study "Preterm SAMBA" which was jointly financed by the Brazilian CNPq (CNPq, Grant 401636/2013-5) and the Bill and Melinda Gates Foundation (Grant OPP1107597).

Competing interests

The author(s) declare that they have not competing interests.

Word count: 2031 words

For peer review only - http://bmjopen.bmj.com/site/about/guidelines.xhtml

Figure Legend

Figure 1. Flow chart of studies identified to be included in the systematic review.

For peer review only

For peer review only - http://bmjopen.bmj.com/site/about/guidelines.xhtml

References

1. Tranquilli AL, Dekker G, Magee L, Roberts J, Sibai BM, Steyn W, et al. The classification, diagnosis and management of the hypertensive disorders of pregnancy: A revised statement from the ISSHP. Pregnancy Hypertens. 2014;4(2):97-104.
2. Brown MA, Magee LA, Kenny LC, Karumanchi SA, McCarthy FP, Saito S, et al. The hypertensive disorders of pregnancy: ISSHP classification, diagnosis & management recommendations for international practice. Pregnancy Hypertens. 2018.
3. Abalos E, Cuesta C, Grosso AL, Chou D, Say L. Global and regional estimates of preeclampsia and eclampsia: a systematic review. Eur J Obstet Gynecol Reprod Biol. 2013;170(1):1-7.
4. Moussa HN, Arian SE, Sibai BM. Management of hypertensive disorders in pregnancy. Women´s Health (Lond). 2014;10(4):385-404.
5. Lotufo FA, Parpinelli MA, Osis MJ, Surita FG, Costa ML, Cecatti JG. Situational analysis of facilitators and barriers to availability and utilization of magnesium sulfate for eclampsia and severe preeclampsia in the public health system in Brazil. BMC Pregnancy Childbirth. 2016;16:254.
6. Myers JE, Kenny LC, McCowan LM, Chan EH, Dekker GA, Poston L, et al. Angiogenic factors combined with clinical risk factors to predict preterm pre-eclampsia in nulliparous women: a predictive test accuracy study. BJOG. 2013;120(10):1215-23.
7. Rolnik DL, Wright D, Poon LC, O'Gorman N, Syngelaki A, de Paco Matallana C, et al. Aspirin versus Placebo in Pregnancies at High Risk for Preterm Preeclampsia. N Engl J Med. 2017;377(7):613-22.
8. Gasse C, Boutin A, Coté M, Chaillet N, Bujold E, Demers S. First-trimester mean arterial blood pressure and the risk of preeclampsia: The Great Obstetrical Syndromes (GOS) study. Pregnancy Hypertens. 2017.
9. Poon LC, Syngelaki A, Akolekar R, Lai J, Nicolaides KH. Combined screening for preeclampsia and small for gestational age at 11-13 weeks. Fetal Diagn Ther. 2013;33(1):16-27.
10. Poon LC, Nicolaides KH. First-trimester maternal factors and biomarker screening for preeclampsia. Prenat Diagn. 2014;34(7):618-27.
11. Cnossen JS, Vollebregt KC, de Vrieze N, ter Riet G, Mol BW, Franx A, et al. Accuracy of mean arterial pressure and blood pressure measurements in predicting pre-eclampsia: systematic review and meta-analysis. BMJ. 2008;336(7653):1117-20.
12. Kenny LC, Black MA, Poston L, Taylor R, Myers JE, Baker PN, et al. Early pregnancy prediction of preeclampsia in nulliparous women, combining clinical risk and biomarkers: the Screening for Pregnancy Endpoints (SCOPE) international cohort study. Hypertension. 2014;64(3):644-52.
13. Parra-Cordero M, Rodrigo R, Barja P, Bosco C, Rencoret G, Sepúlveda-Martinez A, et al. Prediction of early and late pre-eclampsia from maternal characteristics, uterine artery Doppler and markers of vasculogenesis during first trimester of pregnancy. Ultrasound Obstet Gynecol. 2013;41(5):538-44.
14. Kuc S, Koster MP, Franx A, Schielen PC, Visser GH. Maternal characteristics, mean arterial pressure and serum markers in early prediction of preeclampsia. PLoS One. 2013;8(5):e63546.
15. Rodriguez A, Tuuli MG, Odibo AO. First-, Second-, and Third-Trimester Screening for Preeclampsia and Intrauterine Growth Restriction. Clin Lab Med. 2016;36(2):331-51.
16. Ciborowski M, Teul J, Martin-Ventura JL, Egido J, Barbas C. Metabolomics with LC-QTOF-MS permits the prediction of disease stage in aortic abdominal aneurysm based on plasma metabolic fingerprint. PLoS One. 2012;7(2):e31982.

For peer review only - http://bmjopen.bmj.com/site/about/guidelines.xhtml

17. Boudonck KJ, Mitchell MW, Német L, Keresztes L, Nyska A, Shinar D, et al. Discovery of metabolomics biomarkers for early detection of nephrotoxicity. Toxicol Pathol. 2009;37(3):280-92.
18. Balls M. Future improvements: replacement in vitro methods. ILAR J. 2002;43 Suppl:S69-73.
19. Kenny LC. Metabolomics of preeclampsia. Pregnancy Hypertension. 2010;1:S2.
20. Odibo AO, Goetzinger KR, Odibo L, Cahill AG, Macones GA, Nelson DM, et al. First-trimester prediction of preeclampsia using metabolomic biomarkers: A discovery phase study. Prenatal Diagnosis. 2011;31(10):990-4.
21. Bahado-Singh RO, Akolekar R, Mandal R, Dong E, Xia J, Kruger M, et al. Metabolomics and first-trimester prediction of early-onset preeclampsia. Journal of Maternal-Fetal and Neonatal Medicine. 2012;25(10):1840-7.
22. Kenny LC, Broadhurst DI, Dunn W, Brown M, North RA, McCowan L, et al. Robust early pregnancy prediction of later preeclampsia using metabolomic biomarkers. Hypertension. 2010;56(4):741-9.
23. Kuc S, Koster MP, Pennings JL, Hankemeier T, Berger R, Harms AC, et al. Metabolomics profiling for identification of novel potential markers in early prediction of preeclampsia. PLoS One. 2014;9(5):e98540.
24. Wright RW, Brand RA, Dunn W, Spindler KP. How to write a systematic review. Clin Orthop Relat Res. 2007;455:23-9.
25. Liberati A, Altman DG, Tetzlaff J, Mulrow C, Gøtzsche PC, Ioannidis JP, et al. The PRISMA statement for reporting systematic reviews and meta-analyses of studies that evaluate healthcare interventions: explanation and elaboration. BMJ. 2009;339:b2700.
26. Lisonkova S, Joseph KS. Incidence of preeclampsia: risk factors and outcomes associated with early- versus late-onset disease. Am J Obstet Gynecol. 2013;209(6):544.e1-.e12.
27. Whiting PF, Rutjes AW, Westwood ME, Mallett S, Deeks JJ, Reitsma JB, et al. QUADAS-2: a revised tool for the quality assessment of diagnostic accuracy studies. Ann Intern Med. 2011;155(8):529-36.
28. Poon LC, Kametas NA, Chelemen T, Leal A, Nicolaides KH. Maternal risk factors for hypertensive disorders in pregnancy: a multivariate approach. J Hum Hypertens. 2010;24(2):104-10.
29. Austdal M, Skråstad RB, Gundersen AS, Austgulen R, Iversen AC, Bathen TF. Metabolomic biomarkers in serum and urine in women with preeclampsia. PLoS One. 2014;9(3):e91923.
30. Austdal M, Tangerås LH, Skråstad RB, Salvesen KÅ, Austgulen R, Iversen AC, et al. First trimester urine and serum metabolomics for prediction of preeclampsia and gestational hypertension: A prospective screening study. International Journal of Molecular Sciences. 2015;16(9):21520-38.
31. Dessì A, Marincola FC, Fanos V. Metabolomics and the great obstetrical syndromes - GDM, PET, and IUGR. Best Practice and Research: Clinical Obstetrics and Gynaecology. 2015;29(2):156-64.

For peer review only - http://bmjopen.bmj.com/site/about/guidelines.xhtml

For peer review only

For peer review only - http://bmjopen.bmj.com/site/about/guidelines.xhtml

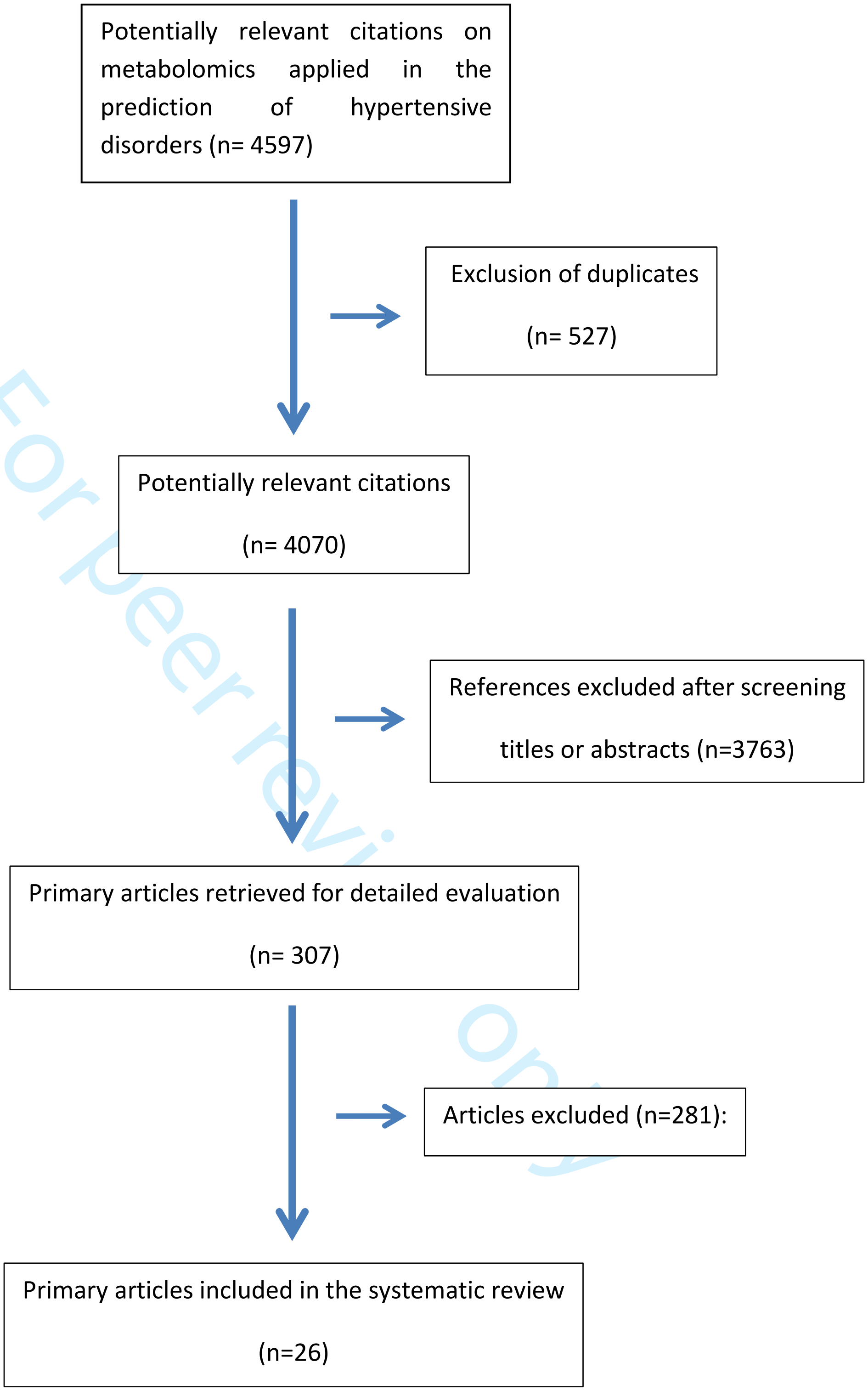

For peer review only

PRISMA-P 2015 Checklist

This checklist has been adapted for use with systematic review protocol submissions to BioMed Central journals from Table 3 in Moher D et al: Preferred reporting items for systematic review and meta-analysis protocols (PRISMA-P) 2015 statement. *Systematic Reviews* 2015 **4**:1

An Editorial from the Editors-in-Chief of *Systematic Reviews* details why this checklist was adapted - **Moher D, Stewart L & Shekelle P:** Implementing PRISMA-P: recommendations for prospective authors. *Systematic Reviews* 2016 **5**:15

Section/topic	#	Checklist item	Information reported Yes	Information reported No	Line number(s)
**ADMINISTRATIVE INFORMATION**					
**Title**					
Identification	1a	Identify the report as a protocol of a systematic review	x		3
Update	1b	If the protocol is for an update of a previous systematic review, identify as such		x	
**Registration**	2	If registered, provide the name of the registry (e.g., PROSPERO) and registration number in the Abstract	x		52
**Authors**					
Contact	3a	Provide name, institutional affiliation, and e-mail address of all protocol authors; provide physical mailing address of corresponding author	x		5,6,7,8
Contributions	3b	Describe contributions of protocol authors and identify the guarantor of the review	x		347-353
**Amendments**	4	If the protocol represents an amendment of a previously completed or published protocol, identify as such and list changes; otherwise, state plan for documenting important protocol amendments		x	
**Support**					
Sources	5a	Indicate sources of financial or other support for the review	x		344-345
Sponsor	5b	Provide name for the review funder and/or sponsor	x		344-345
Role of sponsor/funder	5c	Describe roles of funder(s), sponsor(s), and/or institution(s), if any, in developing the protocol		x	
**INTRODUCTION**					
**Rationale**	6	Describe the rationale for the review in the context of what is already known	x		54-91

BioMed Central The Open Access Publisher

For peer review only - http://bmjopen.bmj.com/site/about/guidelines.xhtml

2

Section/topic	#	Checklist item	Information reported Yes	No	Line number(s)
**Objectives**	7	Provide an explicit statement of the question(s) the review will address with reference to participants, interventions, comparators, and outcomes (PICO)	x☐	☐	94-99
**METHODS**					
**Eligibility criteria**	8	Specify the study characteristics (e.g., PICO, study design, setting, time frame) and report characteristics (e.g., years considered, language, publication status) to be used as criteria for eligibility for the review	x☐	☐	107-109; 128-132; 140-143
**Information sources**	9	Describe all intended information sources (e.g., electronic databases, contact with study authors, trial registers, or other grey literature sources) with planned dates of coverage	x☐	☐	104-107
**Search strategy**	10	Present draft of search strategy to be used for at least one electronic database, including planned limits, such that it could be repeated	x☐	☐	109-114
STUDY RECORDS					
Data management	11a	Describe the mechanism(s) that will be used to manage records and data throughout the review	x☐	☐	121-122
Selection process	11b	State the process that will be used for selecting studies (e.g., two independent reviewers) through each phase of the review (i.e., screening, eligibility, and inclusion in meta-analysis)	x☐	☐	122-126
Data collection process	11c	Describe planned method of extracting data from reports (e.g., piloting forms, done independently, in duplicate), any processes for obtaining and confirming data from investigators	x☐	☐	184-191
**Data items**	12	List and define all variables for which data will be sought (e.g., PICO items, funding sources), any pre-planned data assumptions and simplifications	☐	x☐	
**Outcomes and prioritization**	13	List and define all outcomes for which data will be sought, including prioritization of main and additional outcomes, with rationale	x☐	☐	145-180
**Risk of bias in individual studies**	14	Describe anticipated methods for assessing risk of bias of individual studies, including whether this will be done at the outcome or study level, or both; state how this information will be used in data synthesis	x☐	☐	193-204
DATA					
**Synthesis**	15a	Describe criteria under which study data will be quantitatively synthesized	☐	x☐	
	15b	If data are appropriate for quantitative synthesis, describe planned summary measures, methods of handling data, and methods of combining data from studies, including any planned exploration of consistency (e.g., I^2, Kendall's tau)	☐	x☐	
	15c	Describe any proposed additional analyses (e.g., sensitivity or subgroup analyses, meta-regression)	☐	x☐	
	15d	If quantitative synthesis is not appropriate, describe the type of summary planned	x☐	☐	206-219

For peer review only

BioMed Central
The Open Access Publisher

Section/topic	#	Checklist item	Information reported: Yes	Information reported: No	Line number(s)
**Meta-bias(es)**	16	Specify any planned assessment of meta-bias(es) (e.g., publication bias across studies, selective reporting within studies)	☐	x☐	
**Confidence in cumulative evidence**	17	Describe how the strength of the body of evidence will be assessed (e.g., GRADE)	☐	x☐	

For peer review only

BioMed Central
The Open Access Publisher
